# Methyltransferase‐like 3 suppresses phenotypic switching of vascular smooth muscle cells by activating autophagosome formation

**DOI:** 10.1111/cpr.13386

**Published:** 2022-12-23

**Authors:** Ze‐Min Fang, Shu‐Min Zhang, Hanshen Luo, Ding‐Sheng Jiang, Bo Huo, Xiaoxuan Zhong, Xin Feng, Wenlin Cheng, Yue Chen, Gaoke Feng, Xingliang Wu, Fang Zhao, Xin Yi

**Affiliations:** ^1^ Division of Cardiothoracic and Vascular Surgery Sino‐Swiss Heart‐Lung Transplantation Institute, Tongji Hospital, Tongji Medical College, Huazhong University of Science and Technology Wuhan Hubei China; ^2^ Cardiac Rehabilitation Center Fuwai Hospital CAMS&PUMC Beijing China; ^3^ Key Laboratory of Organ Transplantation, Ministry of Education; NHC Key Laboratory of Organ Transplantation; Key Laboratory of Organ Transplantation Chinese Academy of Medical Sciences Wuhan Hubei China; ^4^ Department of Cardiology Zhongnan Hospital of Wuhan University Wuhan Hubei China; ^5^ Institute of Myocardial Injury and Repair Wuhan University Wuhan China; ^6^ Department of Cardiology Renmin Hospital of Wuhan University Wuhan China

## Abstract

Prevention of neointima formation is the key to improving long‐term outcomes after stenting or coronary artery bypass grafting. RNA N^6^‐methyladenosine (m^6^A) methylation has been reported to be involved in the development of various cardiovascular diseases, but whether it has a regulatory effect on neointima formation is unknown. Herein, we revealed that methyltransferase‐like 3 (METTL3), the major methyltransferase of m^6^A methylation, was downregulated during vascular smooth muscle cell (VSMC) proliferation and neointima formation. Knockdown of METTL3 facilitated, while overexpression of METTL3 suppressed the proliferation of human aortic smooth muscle cells (HASMCs) by arresting HASMCs at G2/M checkpoint and the phosphorylation of CDC2 (p‐CDC2) was inactivated by METTL3. On the other hand, the migration and synthetic phenotype of HASMCs were enhanced by METTL3 knockdown, but inhibited by METTL3 overexpression. The protein levels of matrix metalloproteinase 2 (MMP2), MMP7 and MMP9 were reduced, while the expression level of tissue inhibitor of metalloproteinase 3 was increased in HASMCs with METTL3 overexpression. Moreover, METTL3 promoted the autophagosome formation by upregulating the expression of ATG5 (autophagy‐related 5) and ATG7. Knockdown of either ATG5 or ATG7 largely reversed the regulatory effects of METTL3 overexpression on phenotypic switching of HASMCs, as evidenced by increased proliferation and migration, and predisposed to synthetic phenotype. These results indicate that METTL3 inhibits the phenotypic switching of VSMCs by positively regulating ATG5‐mediated and ATG7‐mediated autophagosome formation. Thus, enhancing the level of RNA m^6^A or the formation of autophagosomes is the promising strategy to delay neointima formation.

## Graphical abstract

The expression of methyltransferase‐like 3 (METTL3) was inhibited by proliferation inducers in vascular smooth muscle cells (VSMCs). Overexpression of METTL3 promotes autophagy‐related 5 (ATG5) and ATG7 protein expression to facilitate autophagosome formation, which subsequently inhibits VSMC proliferation, migration and switching from contractile to synthetic phenotype. Knockdown of either ATG5 or ATG7 largely reversed the inhibitory effects of METTL3 on proliferation, migration and phenotypic switching of VSMCs. These findings indicate that METTL3 may inhibit neointima formation by accelerating the formation of autophagosomes.

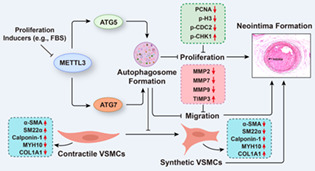

## 1 INTRODUCTION

Neointima formation is one of the main causes of poor prognosis after stenting or coronary artery bypass graft.^1^ It is well known that excessive proliferation of vascular smooth muscle cells (VSMCs) is the major cause of neointima formation. Paclitaxel‐eluting stents and limus‐eluting stents are the two common types of stents coated with anti‐proliferative drugs, which significantly improve the long‐term outcomes of patients after stenting.^2^ However, the challenge of developing more effective drugs remains formidable. The proliferation of VSMCs is regulated by a variety of molecular mechanisms, and our recently published results showed that autophagy is involved in the growth of VSMCs.^3^, ^4^ Moreover, autophagy has also been reported to be participated in the phenotypic switching of VSMCs.^5^ However, the regulatory mechanisms need to be further elucidated.

Various epigenetic modifications have been reported to be contributed to the regulation of VSMC proliferation or autophagy.^6^, ^7^, ^8^ N^6^‐methyladenosine (m^6^A) RNA methylation is an RNA epigenetic modification that occurs in the N6‐position of adenosine, which has been shown to be involved in the occurrence and development of various cardiovascular diseases.^9^ Methyltransferase‐like 3 (METTL3) is the major methyltransferase that mediates RNA m^6^A modification.^9^ Although METTL3 has been studied in atherosclerosis,^10^, ^11^ abdominal aortic aneurysm^12^ and tumour angiogenesis,^13^ it is unknown whether METTL3 regulates VSMC proliferation and neointima formation.

Recently, METTL3 was reported to participate in the regulation of autophagy in cardiomyocytes and tumour cells, but its role in autophagy is still controversial.^14^, ^15^ For example, Song et al.^14^ demonstrated that METTL3 suppressed autophagic flux in cardiomyocytes with hypoxia/reoxygenation treatment, while Liu et al.^15^ showed that METTL3 facilitated autophagy in non‐small cell lung cancer (NSCLC) cells. These results indicate that METTL3 may regulate autophagy in a context‐dependent or cell‐type‐dependent manner. However, it is unclear whether METTL3 affects VSMC phenotypic switching by regulating autophagy. Autophagy has been reported to play an important role in neointima formation and its role is a double‐edged sword. For example, Grootaert et al.^16^ demonstrated that defective autophagy in VSMCs accelerated senescence and promoted neointima formation, whereas Ouyang et al.^17^ showed that SMC‐specific deletion of Ulk1 suppressed autophagy and impeded neointima hyperplasia. Our previous studies also showed that excessive activation of autophagy inhibited VSMC growth and even led to autophagic cell death.^3^, ^4^ Thus, it is imperative to clarify whether METTL3 regulates autophagy, proliferation, as well as phenotypic switching of VSMCs.

In the present study, we found that the expression of METTL3 was negatively correlated with the proliferation of VSMCs. METTL3 inhibited HASMC proliferation by inhibiting the p‐CDC2 and arresting cells at the G2/M checkpoint. METTL3 also suppressed the migration of HASMCs and helped maintain the contractile phenotype of HASMCs. Moreover, METTL3 promoted the expression of autophagy‐related 5 (ATG5) and ATG7, thereby increasing the formation of autophagosomes. Knockdown of either ATG5 or ATG7 to reduce autophagosome formation largely reversed the effects of METTL3 overexpression on VSMCs. Therefore, METTL3 may restrain neointima formation by activating the autophagy in VSMCs.

## 2 METHODS AND MATERIALS

### 2.1 Cell culture and treatments

The primary human aortic smooth muscle cells (HASMCs) were cultured as described previously.^4^, ^18^, ^19^ This study was approved by the Tongji Hospital, Tongji Medical College, Huazhong University of Science and Technology Review Board in Wuhan, China. The aortic tissues were soaked in pre‐chilled DME/F12 medium containing 10% fetal bovine serum (FBS) and 1% antibiotics, and transfer it to the laboratory as soon as possible. Furtherly, the intima and media of aortic tissues were stripped with tweezers under a stereo microscope after removal of blood stains and connective tissue with DME/F12 medium. Then, the media was peeled off layer by layer with micro tweezers, and in the case of ensuring that each layer of the media is thin enough to minimize the stretch to avoid smooth muscle cell damage. The dissected media of the vessels was transferred to cell‐culture flask and cut into small pieces (1 × 1 mm). The tissue pieces are evenly spread on the side wall of the culture flask, and the distance is kept about 2 mm. The flasks were placed upright in a 37°C cell incubator with 5% carbon dioxide to dry for half an hour. After the tissue completely attached to the wall, 8 ml of DME/F12 medium containing 10% FBS was added along the opposite side wall, and the culture flask was placed horizontally to ensure medium can infiltrate the tissue. Long spindle‐shaped smooth muscle cells were observed around the tissue pieces in 1 week. After the cells grew, pay great attention to the growth state of the cells under the microscope, and pass the cells when the growth density reaches 80% for the flowing experiments.

### 2.2 Plasmids construction

The full‐length human METTL3 CDS sequence was amplified by polymerase chain reaction (PCR) and cloned into the pHAGE lentiviral vector with a Flag tag. The primers used to amplify the CDS of METTL3 were as follows: METTL3 forward primer: 5′‐CCGACGCGTGCCACCATGTCGGACACGTGGAG‐3′, METTL3 reverse primer: 5′‐ACGCGTCGACTAAATTCTTAGGTTTAGAGATGATAC‐3′. Double‐strand oligonucleotides of shRNA targeting human METTL3, ATG5 and ATG7 were cloned into pLKO.1 plasmids. The target sequences were: shMETTL3‐1: 5′‐GCTGCACTTCAGACGAATTAT‐3′; shMETTL3‐2: 5′‐GCCAAGGAACAATCCATTGTT‐3′; shATG5: 5′‐CTTTGATAATGAACAGTGAGA‐3′; shATG7: 5′‐GGAGTCACAGCTCTTCCTTAC‐3′.

### 2.3 Western blot analysis

The total protein from HASMCs was extracted by RIPA as previously described.^3^, ^4^ The Pierce™ BCA Protein Assay Kit (23,225, Thermo Fisher Scientific) was used to determine the protein concentration. The protein was denatured at 95°C with 5 × Loading buffer, 20 μg of total protein was loaded and separated by sodium dodecyl sulphate polyacrylamide gel electrophoresis. Then, the protein was transferred to a polyvinylidene fluoride membrane (Millipore, IPVH00010). Furtherly, the membrane was incubated with indicated primary antibody overnight at 4°C after blocked by 5% non‐fat milk. Subsequently, incubating with the peroxidase‐conjugated secondary antibody (Jackson ImmunoResearch Laboratories, 111‐035‐003, at 1:25,000 dilution), the protein signals were detected by using the ChemiDocTM XRS^+^ system (Bio‐Rad). The antibodies used in this study including anti‐proliferating cell nuclear antigen (PCNA; GTX100539; 1:1000), anti‐MMP9 (GTX100458; 1:1000), anti‐MMP2 (GTX634832; 1:1000) and anti‐MYH10 (GTX634160; 1:1000) are obtained from GeneTex. Anti‐METTL3 (15073‐1‐AP; 1;1000) and anti‐tissue inhibitor of metalloproteinase 3 (TIMP3; 10858‐1‐AP; 1:1000) get from Proteintech Group. Anti‐β‐actin (no. 8457 S; 1:1000), anti‐p‐CDC2 (no. 4539; 1:1000), anti‐p‐CHK1 (no. 2348; 1:1000), anti‐p‐CHK2 (no. 2197; 1:1000), anti‐CDK4 (no. 12790; 1:1000), anti‐cyclinD3 (no. 2936; 1:1000), anti‐Calponin‐1 (no. 1264; 1:1000), anti‐COL1A1 (no. 91144; 1:1000), anti‐ATG5 (no. 12994; 1:1000), anti‐ATG7 (no. 8558; 1:1000), anti‐phosphorylation of mammalian target of rapamycin (p‐mTOR; no. 5536; 1:1000), anti‐mTOR (no. 2983; 1:1000), anti‐P21 (no. 2947; 1:1000), anti‐SQSTM1 (no. 95697; 1:1000), anti‐phosphorylated ULK1 (p‐ULK1; no. 5869; 1:1000) and anti‐LC3 (no. 12741; 1:1000) obtained from Cell Signalling Technology. Anti‐MMP7 (ab205525; 1:1000), anti‐α‐smooth muscle actin (α‐SMA; ab7817; 1:200) and anti‐SM22α (ab14106; 1:1000) acquired from Abcam, and anti‐p‐Histone H3 (sc‐8656‐R; 1:200) get from Santa Cruz.

### 2.4 Real‐time PCR

Real‐time PCR was performed as previously described.^3^, ^20^ Total mRNA was extracted with TRI Reagent® Solution (A33251, Invitrogen), and 5 μg of total mRNA was reverse transcription into cDNA by using Hifair® II first Strand cDNA Synthesis Kit (gDNA digester plus; 11119ES60, Yeasen). Then, the mRNA levels of target genes were detected by using Hifair® qPCR SYBR® Green Master Mix (No Rox; 11201ES08, Yeasen) in CFX connect™ real‐time PCR detection system (Bio‐Rad). Primers used in this study were as follow: METTL3 forward primer 5′‐TGGGGGTATGAACGGGTAGA‐3′ and reverse primer 5′‐TGGTTGAAGCCTTGGGGATT‐3′.

### 2.5 EdU incorporation assay

5‐ethynyl‐2'‐deoxyuridine (EdU) incorporation assay was performed as previously described.^21^ Briefly, a Cell‐Light™ Edu Apollo567 In Vitro kit (C10310‐1, RiboBio) was used to perform the EdU incorporation assay. After overexpressing or knocking down of METTL3, HASMCs were seeded into 24‐well plates at 3 × 10^4^ cells per well. After incubating with 50 μM EdU medium (300 μl per well) for 2 h, the cells were fixed with 4% paraformaldehyde for 30 min and incubated with 2 mg/ml glycine for 5 min to neutralize paraformaldehyde. Cells were incubated with 1× Apollo staining solution for 30 min at room temperature in the dark after washing with phosphate‐buffered saline (PBS) containing 0.5% Triton X‐100 for 10 min. Then, washing the cells with 0.5% Triton X‐100 PBS solution again, and cells were incubated with 1× Hoechst 33342 for 30 min at room temperature in the dark. Last, cells were washed with PBS for three times. The fluorescence microscope was used to collect fluorescence images of cells.

### 2.6 Immunofluorescence assay

The porcine model of restenosis after stenting was generated as our previously described.^22^, ^23^ After porcine were sacrificed, the coronary arteries were removed from the heart and fixed with 10% formalin, then paraffin embedded and sectioned into 5 μm. The sections were then stained with haematoxylin–eosin, and observed under the microscope. The expression and localization of METTL3 were determined according to a previously described immunofluorescence method.^3^, ^4^ Slides with tissue sections were dewaxed with paraformaldehyde and gradually hydrated with ethanol. Then, the slides were placed in EDTA repair solution at 100°C for 20 min. After the slides were blocked with blocking buffer (5% bovine serum albumin) at 37°C for 30 min, the primary antibody METTL3 (Proteintech 15,073‐1‐AP, 1:200 dilution) and α‐SMA (Abcam, ab7817, 1:200 dilution) were incubated for overnight at 4°C. Subsequently, the secondary antibodies (Alexa Fluor 568 donkey anti‐Rabbit IgG [H + L; Thermo Fisher Scientific, A10042] for METTL3 and the Alexa Fluor 488 donkey anti‐mouse IgG [H + L; Thermo Fisher Scientific, A21202] for α‐SMA) were incubated for 60 min at 37°C and followed with diamidino‐phenyl‐indole (DAPI) staining in the dark. After washing with PBS, an Olympus light microscope BX53 system was applied for images capture.

### 2.7 Detection of autophagic flux

The autophagic flux was dynamically observed in HASMCs with mCherry‐GFP‐LC3B overexpression as described previously.^14^ To further evaluate the effect of METTL3 on autophagic flux, we further knocked down or overexpressed METTL3 in mCherry‐GFP‐LC3B‐overexpressing HASMCs. Chloroquine (CQ) (20 μM; C6628; Sigma‐Aldrich), a lysosomotropic agent, was used to inhibit the content degradation of autolysosome. After treated with indicated stimulus, the HASMCs were fixed with 4% paraformaldehyde in PBS for 10 min. The fluorescence images were acquired by using a fluorescence microscope. Yellow and red colour indicate autophagosomes or autolysosomes, respectively.

### 2.8 Cell viability assay

A cell counting kit‐8 (CCK‐8) assay (CK04, Dojindo, Kumamoto, Japan) was used to assess the cell viability of HASMCs. HASMCs infected with lenti‐shMETTL3 or lenti‐METTL3 or their control lentivirus, and seeded in 96‐well plates at a concentration of 8 × 10^3^ cells per well. Then, washing with sterile PBS, cells were incubated with WST‐8 [2‐(2‐methoxy‐4‐nitrophenyl)‐3‐(4‐nitrophenyl)‐5‐(2,4‐disulfophenyl)‐2H‐tetrazolium, monosodium salt] for 2 h. The microplate reader (BioTek Instruments) was used to measure the optical density value of absorbance at 450 nm.

### 2.9 Transwell assay

After starving with serum‐free medium for 24 h, the HASMCs were digested and resuspend in 0.5% FBS medium. A total of 100 μl of cell suspension (3 × 10^4^) was added to the upper chambers of a transwell culture plate. After the cells adhered for 2.5 h, 800 μl of medium containing 10% FBS was added to the bottom chamber. The plate was incubated in cell incubator with 5% CO_2_ for 12 h. After that, discarding the culture medium in the well, and the cells were fixed with 4% paraformaldehyde for 15 min and then stained with 0.1% crystal violet for 30 min. The non‐migrated cells on the upper surface of the well were carefully removed with wet cotton swabs. Then, the cells were observed with a microscope.

### 2.10 Flow cytometry

After treated with indicated stimulus, the HASMCs were collected by trypsin digestion and then washed with PBS for twice. After centrifugation for 10 min, the cells were resuspended with 500 μl PBS, then 5 ml pre‐cooled ethanol was added and put it at 4°C overnight. After discarding the ethanol, the cells were washed twice with PBS. Finally, the cells were treated with 0.3 mg of Ribonuclease A (R5125; Sigma‐Aldrich) and stained by 0.015 mg of PI (P4864; Sigma‐Aldrich) for 2 h in the dark. A BD FACS Aria™ III sorter was used to analyse the cycle of the target cells.

### 2.11 Statistical analyses

The data were analysed by GraphPad Prism 9 software in this study. All the results were represented as mean ± SD. Student's *t*‐test was used to analyse the means of two groups. Multiple group comparisons were performed by using one‐way ANOVA with post hoc analysis. A *p*‐value < 0.05 is considered to be statistically significant.

## 3 RESULTS

### 3.1 METTL3 was downregulated during VSMC proliferation

**FIGURE 1:**
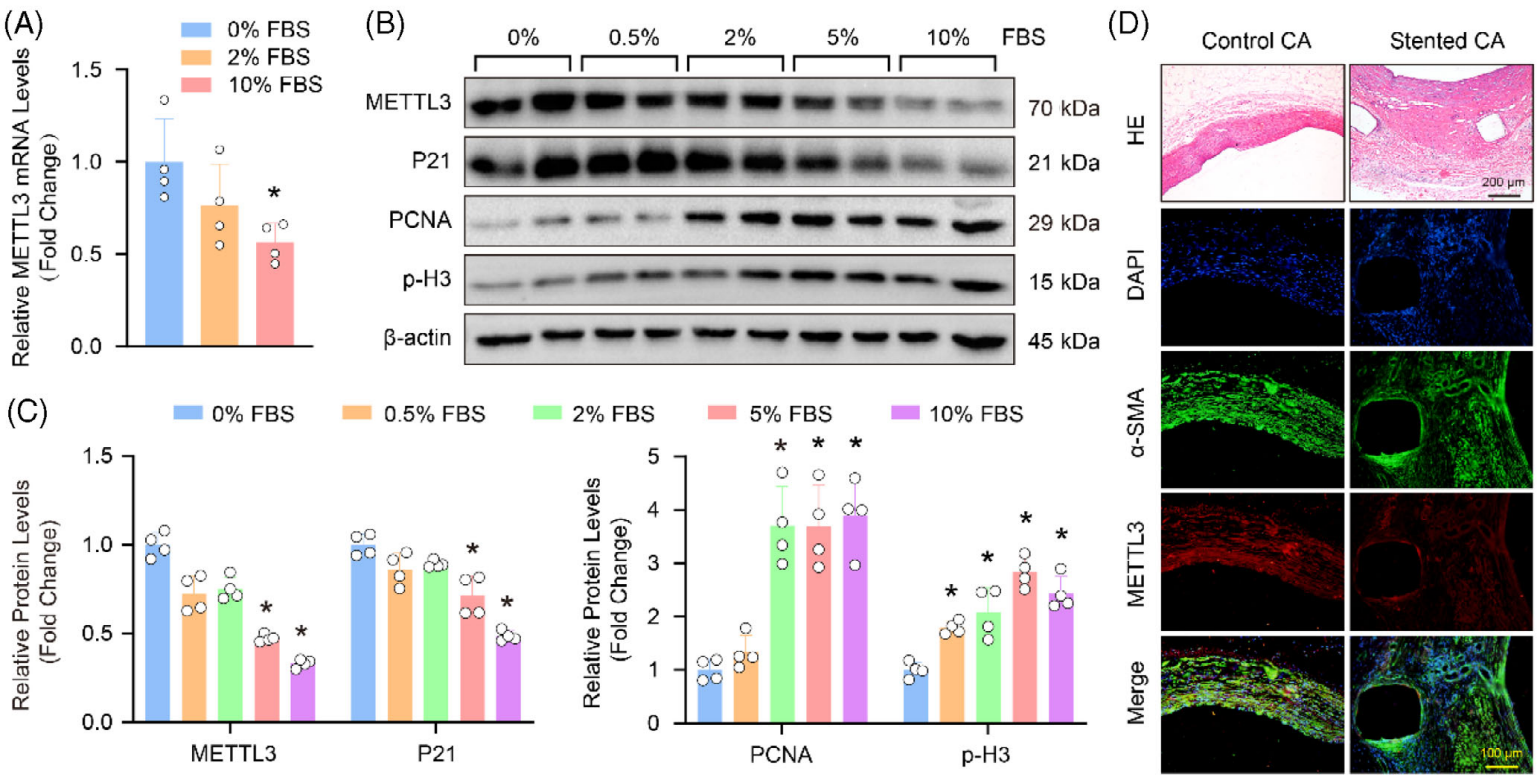
The expression level of methyltransferase‐like 3 (METTL3) reduced during vascular smooth muscle cell (VSMC) proliferation and neointima formation. (A) The mRNA levels of METTL3 was detected by RT‐PCR in human aortic smooth muscle cells (HASMCs) with 0%, 2% or 10% fetal bovine serum (FBS) treatment (*n* = 4). (B,C) The protein levels of METTL3, P21, PCNA and p‐H3 were evaluated by western blots in HASMCs with different concentrations of FBS stimulation; (B) Representative blots; (C) Quantitative results (*n* = 4). β‐Actin serves as loading control. (D) The haematoxylin–eosin (HE) staining (scale bar: 200 μm) And immunofluorescence staining (scale bar: 100 μm) In coronary arteries (CAs) with or without stent implantation. Blue staining is nucleus (DAPI positive); Green staining is VSMCs (α‐SMA positive); Red fluorescence indicates positive METTL3 staining (*n* = 3). *p < 0.05 versus 0% FBS

To investigate whether METTL3 was involved in the process of VSMC proliferation, the HASMCs were treated with different concentrations of FBS to induce proliferation. We found that compared with FBS‐free group, the mRNA level of METTL3 was obviously decreased in HASMCs treated with 10% FBS (Figure 1A). Similarly, the protein level of METTL3 was also significantly down‐regulated in HASMCs with 5% and 10% FBS, and the markers of proliferation, PCNA (proliferating cell nuclear antigen) and p‐H3 (phosphorylation of histone H3) were robustly elevated in HASMCs with 2%, 5% and 10% FBS treatment, while the expression level of P21 significantly reduced (Figure 1B,C). In addition, we generated a pig model of restenosis after coronary stenting.^22^ Our results showed that obvious coronary stenosis with smaller lumen was observed after stenting (Figure 1D). However, METTL3 was downregulated in the stented coronary arteries (Figure 1D). These results indicated that METTL3 may involve in the proliferation of VSMCs and neointima formation.

### 3.2 METTL3 inhibited the proliferation of HASMCs

**FIGURE 2:**
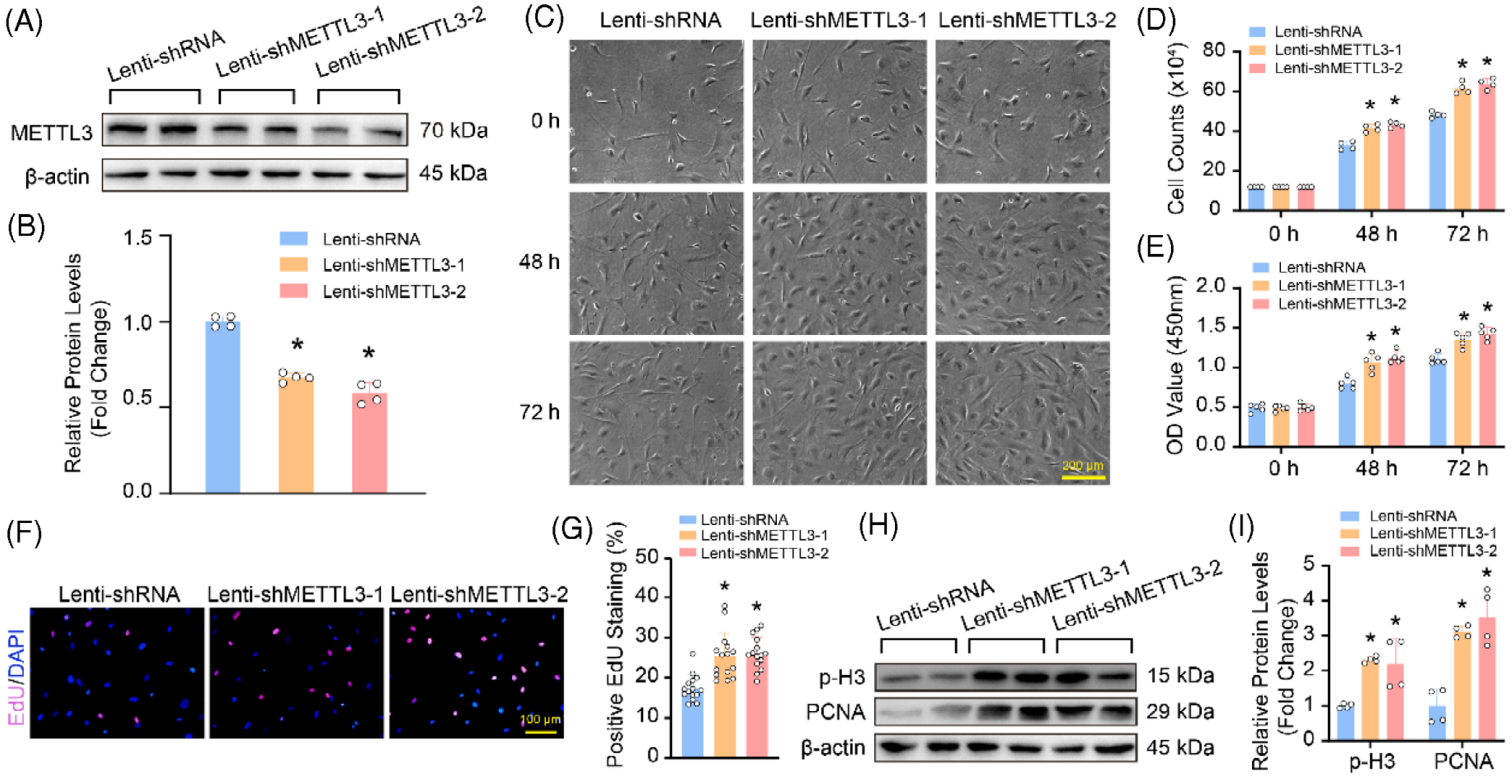
The proliferation of human aortic smooth muscle cells (HASMCs) was enhanced by methyltransferase‐like 3 (METTL3) knockdown. (A,B) The efficiency of METTL3 knockdown was verified by western blot (*n* = 4); (A) Representative blots; (B) Quantitative results. β‐Actin serves as loading control. (C) Representative HASMC images under the light microscope at different time point, Scale bar: 200 μm. (D) HASMC numbers were counted at indicated time points after knocking down METTL3 or not (*n* = 4). (E) The cell viability was evaluated by CCK8 assay at indicated time points, and absorbance was detected at 450 nm wavelength (*n* = 5). (F,G) EdU incorporation assay was performed for monitoring cell proliferation; (F) Pink indicates EdU positive staining, and blue indicates DAPI, scale bar: 100 μm; (G) Quantitative result of the percentage of EdU positive staining in each group (*n* = 15). (H,I) The protein levels of proliferation markers, PCNA and p‐H3 were detected by using western blot (*n* = 4); (H) Representative blots; (I) Quantitative results. β‐Actin serves as loading control. *p < 0.05 versus lenti‐shRNA

To investigate the role of METTL3 on VSMC proliferation, we first knockdown of METTL3 in HASMCs by infecting with lentiviruses containing targeting sequences (Figure 2A,B). The result of the growth curve showed that compared with lenti‐shRNA, the cell counts of lenti‐shMETTL3‐1 and lenti‐shMETTL3‐2 groups were obviously increased at 48 and 72 h (Figure 2C,D). The result of CCK8 assay demonstrated that the cell viability was also enhanced by METTL3 knockdown (Figure 2E). EdU incorporation assay was performed to monitor cell division, and more EdU‐positive cells were detected in HASMCs with METTL3 deficiency (Figure 2F,G). Finally, the expression levels of proliferation markers were evaluated, and the results showed that compared with lenti‐shRNA, the protein levels of PCNA and p‐H3 were increased in HASMCs with lenti‐shMETTL3‐1 or lenti‐shMETTL3‐2 treatment (Figure 2H,I).

**FIGURE 3:**
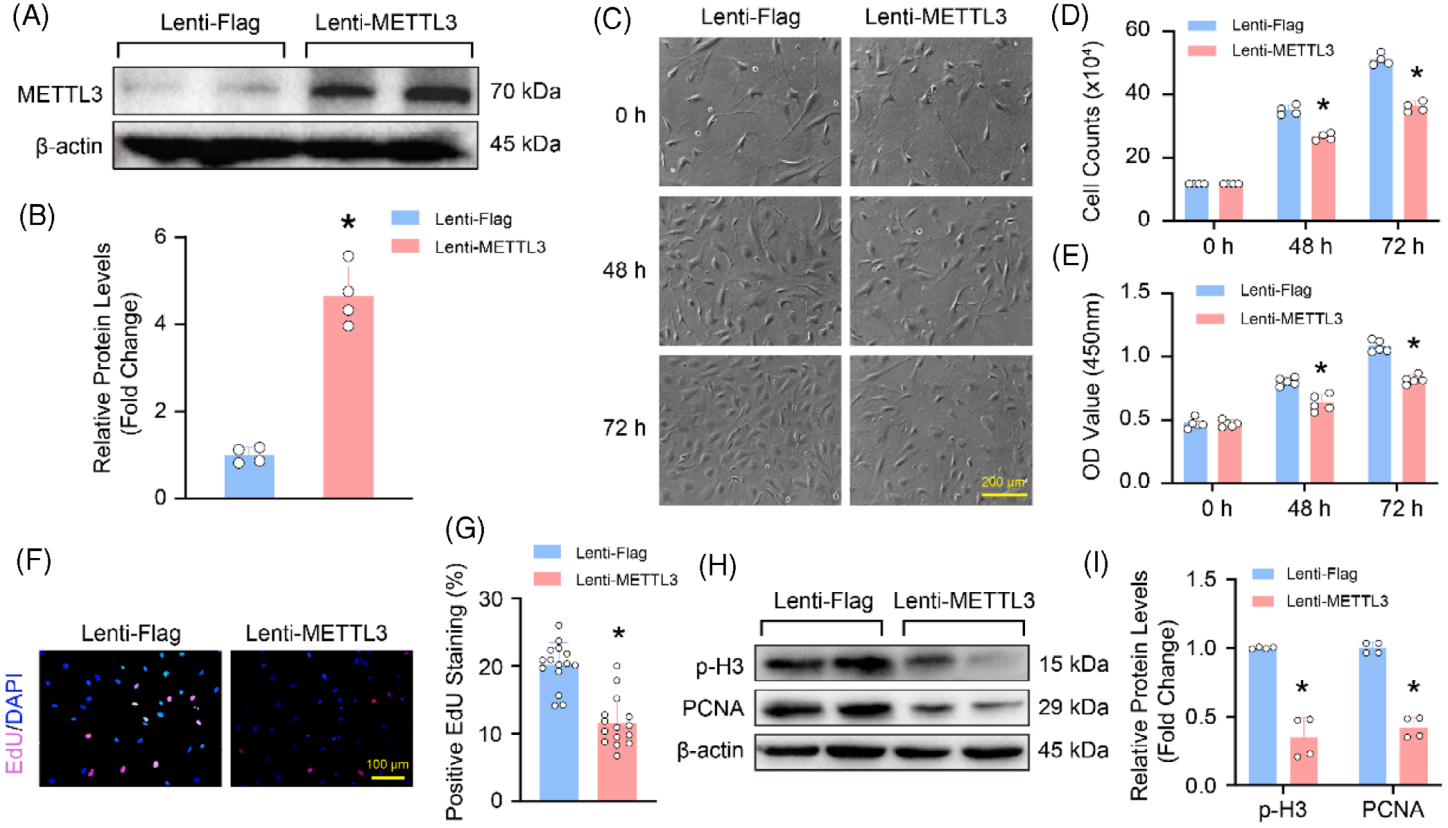
Methyltransferase‐like 3 (METTL3) overexpression suppressed human aortic smooth muscle cell (HASMC) proliferation. (A) Representative blot of METTL3 in HASMCs with or without METTL3 overexpression (*n* = 4); (B) Quantitative results. β‐Actin serves as loading control. (C) Representative HASMC images under the light microscope after METTL3 overexpression or not for indicated times, Scale bar: 200 μm; (D) The counts of HASMCs at indicated time points (*n* = 4). (E) CCK8 assay was used to evaluate the cell viability (*n* = 5). (F,G) EdU incorporation assay indicated that METTL3 overexpression inhibited HASMCs proliferation; (F) Pink: Edu positive staining, blue: DAPI, scale bar: 100 μm; (G) Quantitative result (*n* = 15). Representative blots (H) and quantitative result (I) of PCNA and p‐H3 in HASMCs with METTL3 overexpression or not (*n* = 4). β‐Actin serves as loading control. *p < 0.05 versus lenti‐Flag.

In contrast, METTL3 overexpression significantly inhibited HASMC proliferation (Figure 3A–I), as evidenced by reduced cell numbers (Figure 3C,D), cell viability (Figure 3E), EdU‐positive cells (Figure 3F,G), and decreased PCNA and p‐H3 expression levels (Figure 3H,I) in HASMCs with METTL3 overexpression compared with lenti‐Flag group. Therefore, these results demonstrated that METTL3 negatively regulated HASMC proliferation.

### 3.3 METTL3 arrested HASMCs at G2/M checkpoint

**FIGURE 4:**
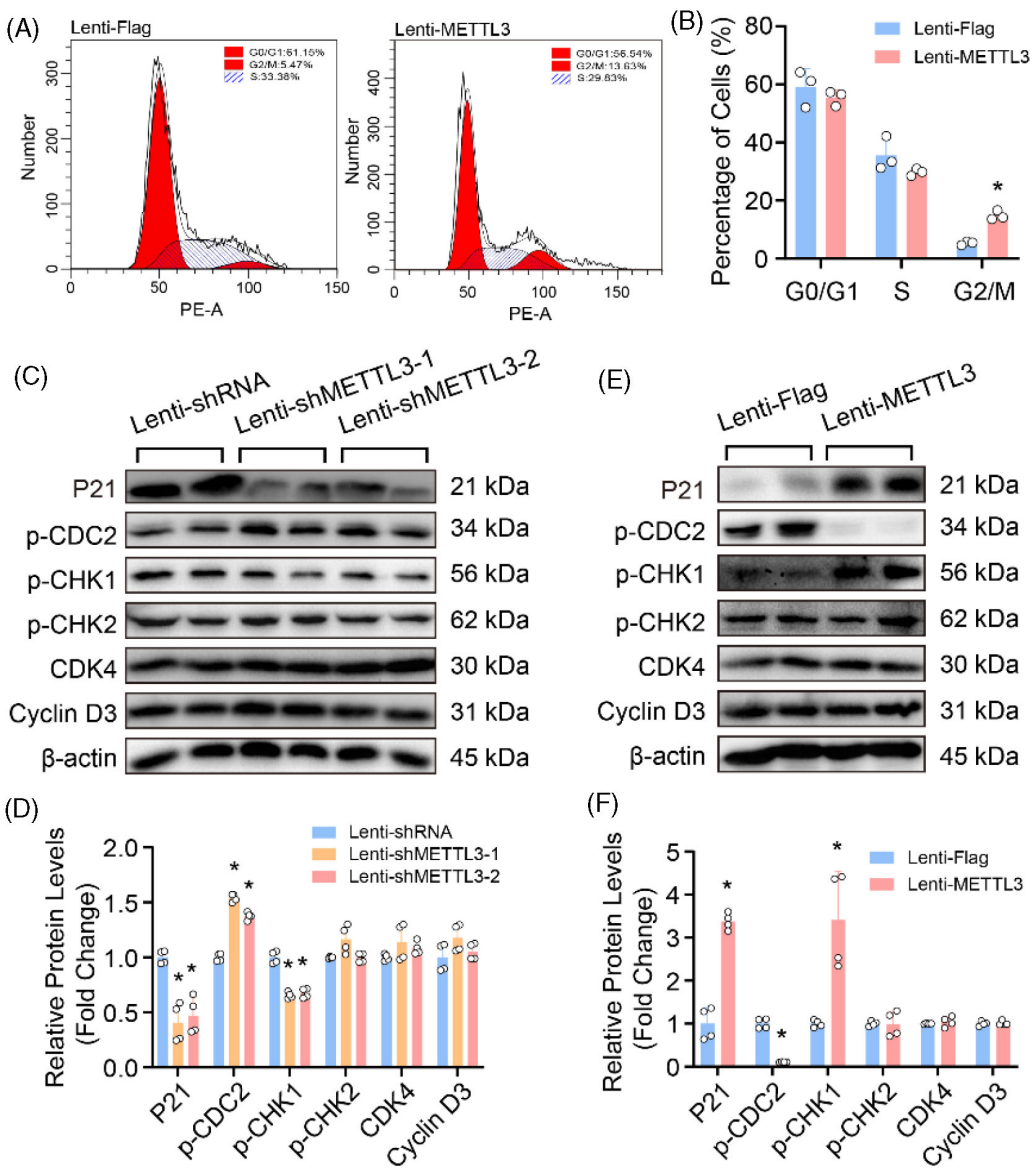
Methyltransferase‐like 3 (METTL3) inhibited the phosphorylation of CDC2 (p‐CDC2) to arrest human aortic smooth muscle cells (HASMCs) at G2/M checkpoint. (A) Representative flow cytometry plots of HASMCs infected with lenti‐METTL3 or lenti‐Flag which were stained with propidium iodide; (B) Quantification of the proportion of cells at each checkpoint in cell cycle (*n* = 3). (C–F) The protein levels of P21, phosphorylation of CDC2 (p‐CDC2), p‐CHK1, p‐CHK2, CDK4 and Cyclin D3 were detected by using western blot in HASMCs with METTL3 knockdown (C) or overexpression (E); Quantitative results of indicated protein levels in METTL3 knocked down HASMCs (D) or METTL3 overexpressed HASMCs (F). β‐Actin serves as loading control. *p < 0.05 versus lenti‐shRNA or lenti‐Flag

To decipher which cell phase regulated by METTL3 affected proliferation, the cell cycle was detected by using flow cytometry in HASMCs with METTL3 overexpression. The result showed that overexpression of METTL3 resulted in more cells arresting at G2/M phase, suggesting that METTL3 is likely to negatively regulate G2/M checkpoint (Figure 4A,B). As we known, the G2/M checkpoint is controlled by CDC2 and Cyclin B,^24^ we found that the p‐CDC2 was obviously downregulated by METTL3 overexpression, but upregulated in HASMCs with METTL3 knockdown (Figure 4C–F). In addition, P21, checkpoint kinase 1 (CHK1) and CHK2 were reported to involve in G2/M transition, and inhibited cell cycle.^25^ Thus, their protein levels were determined by using western blot, and the results demonstrated that METTL3 deficiency suppressed, while overexpression of METTL3 promoted the protein levels of p21 and p‐CHK1 in HASMCs, while neither METTL3 knockdown nor overexpression affected the expression of p‐CHK2, CDK4 and Cyclin D3 (Figure 4C–F). These results suggested that METTL3‐inhibited G2/M transition by downregulating p‐CDC2, while upregulating P21 and p‐CHK1 in HASMCs.

### 3.4 METTL3 repressed phenotypic switching of HASMCs

**FIGURE 5:**
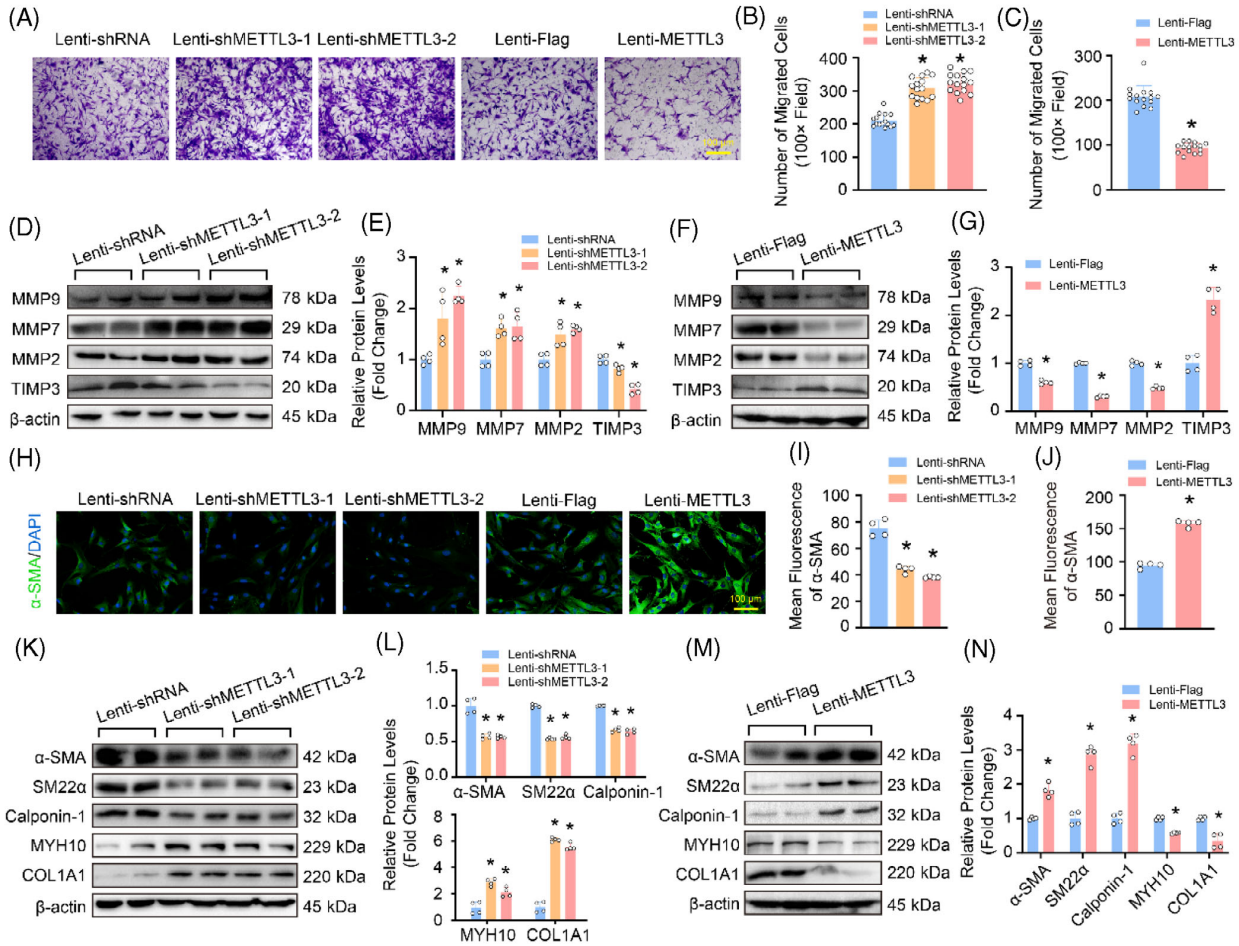
Methyltransferase‐like 3 (METTL3) Contributed to maintaining the contractile phenotype of human aortic smooth muscle cells (HASMCs). (A–C) Transwell assay were used to assess the migratory capacity of HASMCs with METTL3 knockdown or overexpression, and the cells were stained with crystal violet (*n* = 15). (A) Representative images under the light microscope; (B,C) Counting of migrated HASMCs under 100× field. (D–G) The protein levels of matrix metalloproteinase 9 (MMP9), MMP7, MMP2 and tissue inhibitor of metalloproteinase 3 (TIMP3) were detected by using western blot in HASMCs with METTL3 knockdown (D) or overexpression (F; *n* = 4); Quantitative results of indicated protein levels in METTL3 knocked down HASMCs (E) or METTL3 overexpressed HASMCs (G). (H–J) The immunofluorescence staining of α‐SMA in HASMCs with METTL3 knockdown or overexpression (*n* = 4); (H) Representative images, and green indicates α‐SMA positive staining, scale bar: 100 μm; (I,J) Quantitative results of mean fluorescence of α‐SMA. (K–N) Representative blots (K,M) and quantitative results (L,N) of phenotypic switching markers, α‐SMA, SM22a, Calponin‐1, MYH10 and COL1A1 in HASMCs with METTL3 knockdown or overexpression (*n* = 4). β‐Actin serves as loading control. *p < 0.05 versus lenti‐shRNA or lenti‐Flag

It is known that inhibition of VSMC proliferation is often accompanied by changes in phenotypic switching.^26^ As we have shown that METTL3 inhibits HASMC proliferation, we were therefore very curious whether METTL3 also affects the phenotypic switching of HASMCs. We first examined the effects of METTL3 on HASMC migration which was evaluated by transwell assay. The results showed that compared with control, METTL3 knockdown remarkably promoted the migration of HASMCs, while the migration rate of METTL3‐overexpressed HASMCs was significantly reduced (Figure 5A–C). Matrix metalloproteinases (MMPs) and TIMPs are the key regulators of VSMC migration.^19^, ^27^ Our results revealed that the protein levels of MMP2, MMP7 and MMP9 were obviously accelerated by METTL3 deficiency, but inhibited by METTL3 overexpression in HASMCs (Figure 5D–G). However, TIMP3 was upregulated by METTL3 in HASMCs (Figure 5D–G).

It is reported that the phenotypic switching of VSMCs is characterized by decreased expression levels of contractile markers but increased expression levels of synthetic markers, which contributes to VSMC proliferation and migration.^28^ Thus, we further evaluated the effects of METTL3 on phenotypic switching of HASMCs. The result of cellular immunofluorescence showed that the expression of α‐SMA (a contractile marker) was significantly reduced in HASMCs with METTL3 knockdown, while overexpression of METTL3 promoted the expression of α‐SMA (Figure 5H–J). The protein levels of contractile markers, α‐SMA, SM22α and Calponin‐1, were upregulated in HASMCs with METTL3 overexpression, but downregulated in HASMCs with METTL3 knockdown (Figure 5K–N). In contrast, the synthetic markers MYH10 and COL1A1 showed an opposite expression pattern to that of the contractile markers (Figure 5K–N). Therefore, these results indicate that METTL3 negatively regulates migration and switching from contractile to synthetic phenotype of HASMCs.

### 3.5 METTL3 facilitated autophagosome formation in HASMCs

**FIGURE 6:**
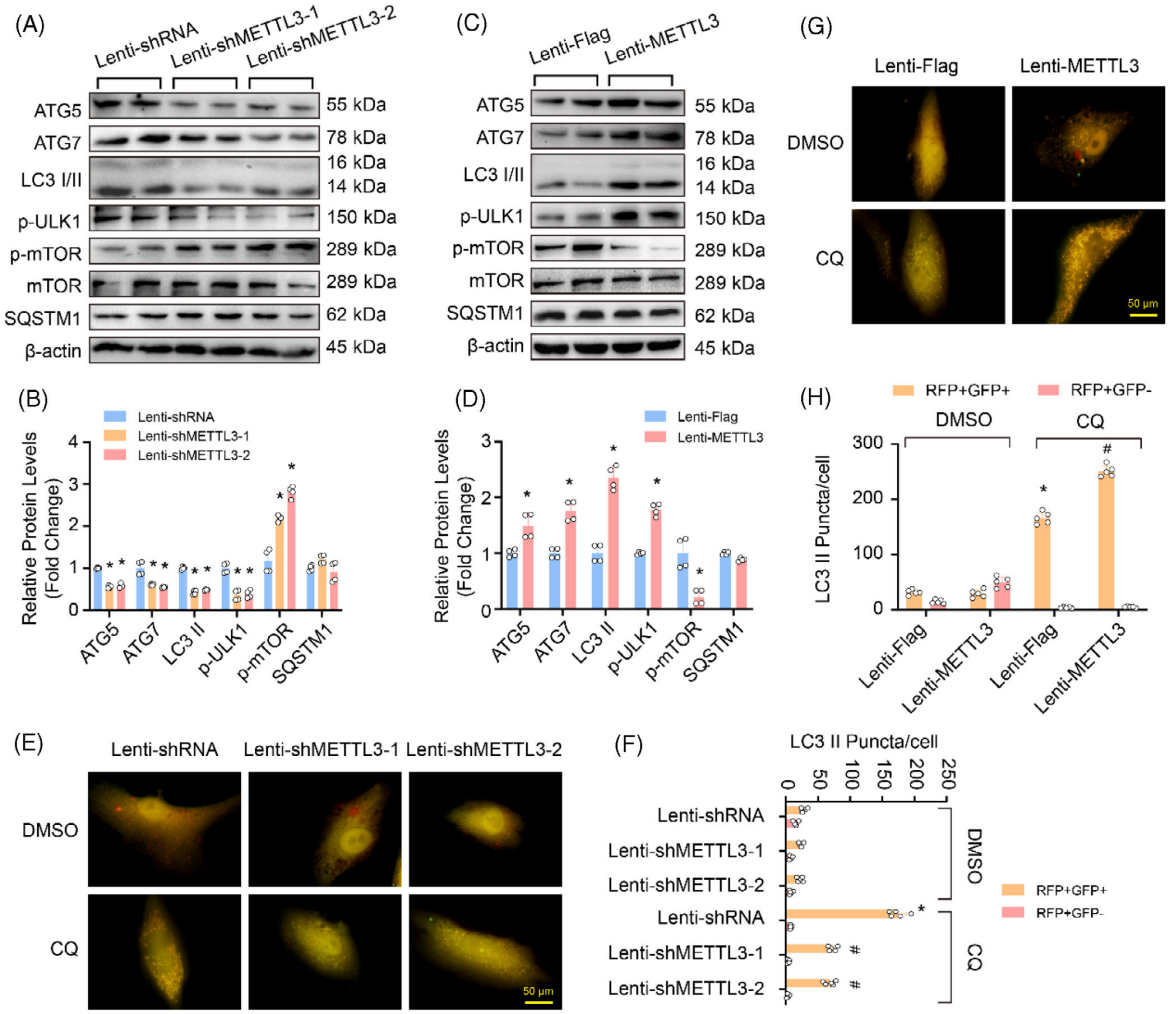
Methyltransferase‐like 3 (METTL3)‐activated autophagy by promoting the expression of autophagy‐related 5 (ATG5) and ATG7. (A–D) Western blot was performed to detect the protein levels of ATG5, ATG7, LC3, phosphorylated ULK1 (p‐ULK1), phosphorylation of mTOR (p‐mTOR) and SQSTM1 in human aortic smooth muscle cells (HASMCs) with METTL3 knockdown or overexpression (*n* = 4); (A,C) Representative blots; (B,D) Quantitative results. β‐Actin serves as loading control. (E–H) The autophagic flux was monitored by mCherry‐GFP‐LC3 overexpression in the HASMCs with indicated treatments; Yellow and red indicate autophagosomes or autolysosomes, respectively. (E,G) Representative images, scale bar: 100 μm, (E) METTL3 knockdown, (G) METTL3 overexpression. (F,H) Quantitative results (*n* = 5). *p < .05 versus lenti‐shRNA or lenti‐Flag. #*p <* 0.05 versus lenti‐shRNA + CQ or lenti‐Flag + CQ

METTL3 is known to regulate autophagy, but its effect on autophagy remains controversial.^14^, ^15^ Therefore, we first examined whether METTL3 regulates autophagy in HASMCs and showed that METTL3 knockdown inhibited the protein levels of ATG5, ATG7, LC3II and p‐ULK1, while enhanced the p‐mTOR (Figure 6A,B). On the contrary, METTL3 overexpression significantly promoted the expression of ATG5, ATG7, LC3II and p‐ULK1, but suppressed p‐mTOR level (Figure 6C,D). These results indicated that METTL3 might enhance autophagy by regulating the expression of multiple molecules involving in autophagy initiation.

As autophagy is a multistep biological process,^8^ thus, to further verify which step was affected by METTL3, the autophagic flux was monitored with mRFP‐GFP‐LC3 assay.^4^ Our results showed that compared with lenti‐shRNA group, both the numbers of orange puncta (autophagosome) and red puncta (autolysosome) were obviously reduced in HASMCs with METTL3 deficiency (Figure 6E,F). Moreover, under the treatment of CQ which is a lysosomotropic agent commonly used to inhibit the content degradation of autolysosome,^3^ more orange puncta were found in the HASMCs with lenti‐shRNA than in the HASMCs with METTL3 knockdown (Figure 6E,F). In contrast, compared with the lenti‐Flag group, the numbers of autophagosomes and autolysosomes were significantly increased in METTL3‐overexpressing HASMCs, and the number of autophagosomes further accumulated after CQ treatment (Figure 6G,H). Therefore, these results indicate that METTL3 remarkably accelerates the formation of autophagosome in HASMCs.

### 3.6 Inhibition of autophagosome formation largely reversed the effects of METTL3 on HASMCs

**FIGURE 7:**
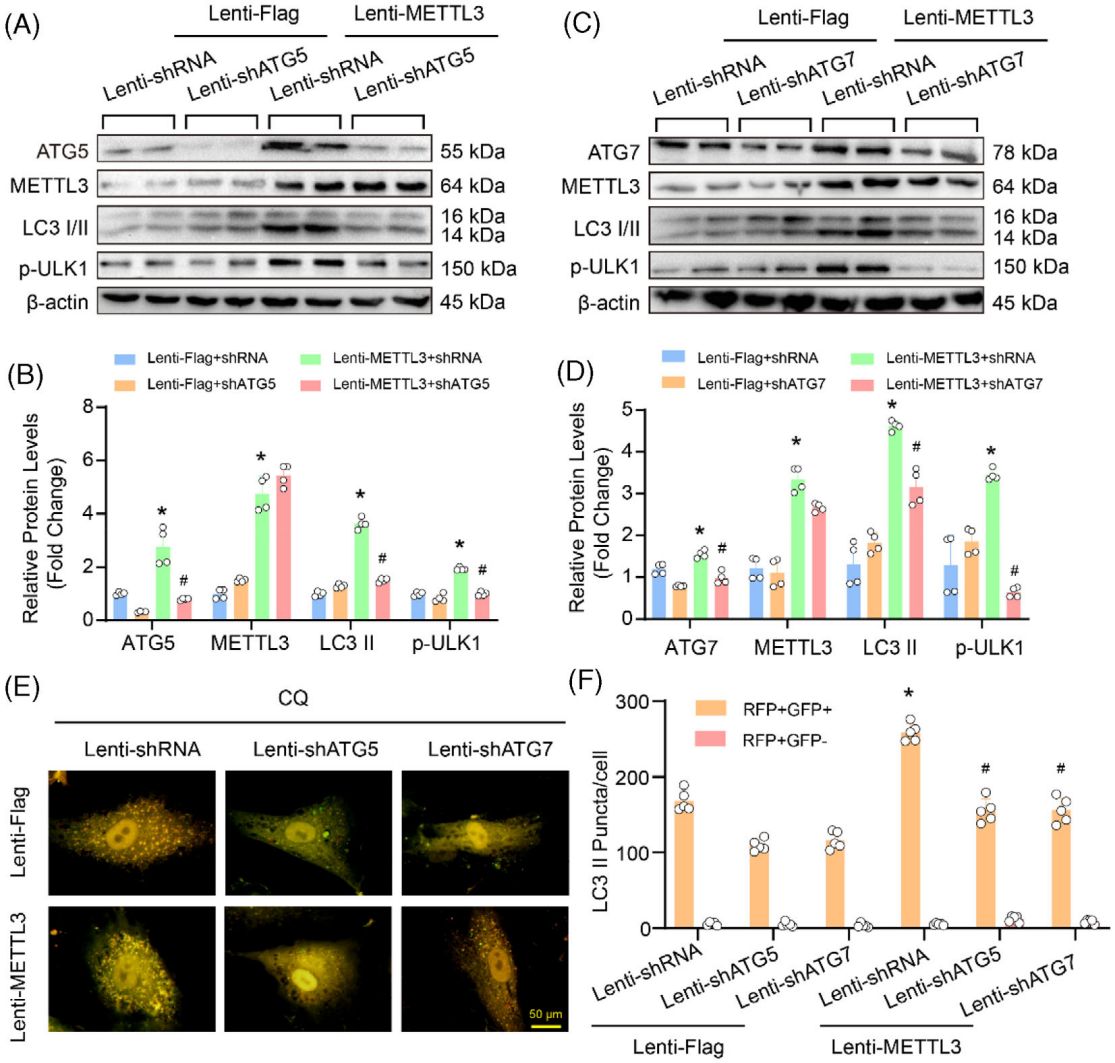
Knockdown of either autophagy‐related 5 (ATG5) or ATG7 reversed the effects of methyltransferase‐like 3 (METTL3) on autophagy activation in human aortic smooth muscle cells (HASMCs). (A–D) The protein levels of ATG5, ATG7, METTL3, LC3 and phosphorylated ULK1 (p‐ULK1) were detected by using western blot in HASMCs with indicated treatments; ATG5 knockdown (A) or ATG7 knockdown (C); quantitative results of indicated protein levels (B,D; *n* = 4). β‐Actin serves as loading control. (E,F) Autophagy flux was detected in mCherry‐GFP‐LC3 overexpressing HASMCs with indicated treatments. Yellow and red indicate autophagosomes or autolysosomes, respectively. (E) Representative images, scale bar: 100 μm, (F) Quantitative results (*n* = 5). *p < 0.05 versus lenti‐Flag + lenti‐shRNA. #*p <* 0.05 versus lenti‐METTL3 + lenti‐shRNA

To determine whether autophagy activation plays a causative role in the METTL3‐mediated inhibition of proliferation, migration and phenotypic switching in HASMCs, ATG5 and ATG7 were knocked down in the HASMCs with METTL3 overexpression (Figure 7A–D). Consistent with previous reports,^3^ ATG5 or ATG7 knockdown significantly suppressed the protein levels of LC3II and p‐ULK1 induced by METTL3 overexpression (Figure 7A–D). Moreover, autophagic flux assay showed that either ATG5 or ATG7 knockdown largely nullified the autophagosome formation induced by METTL3 overexpression in HASMCs (Figure 7E,F).

**FIGURE 8:**
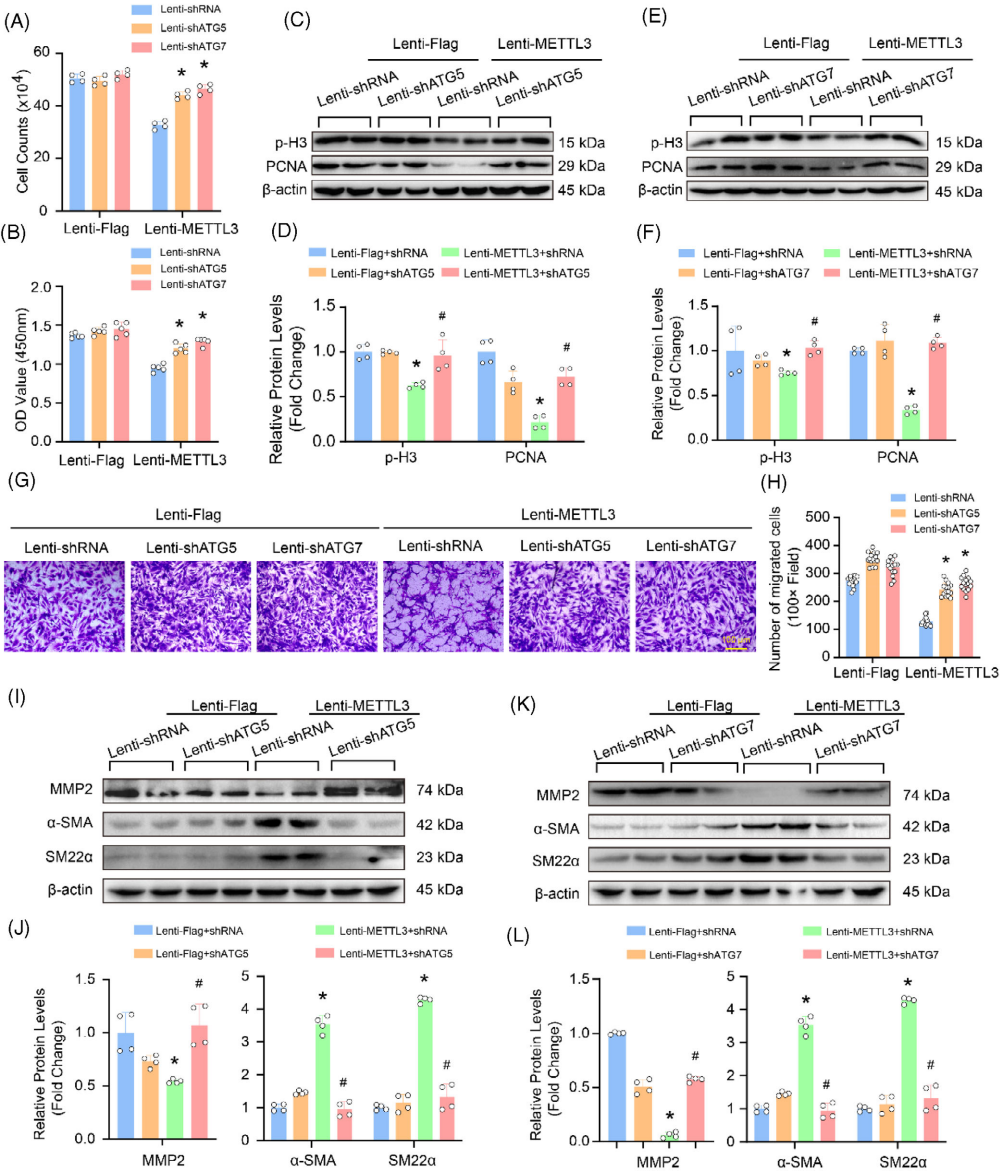
Inhibition of proliferation, migration and phenotypic switching by methyltransferase‐like 3 (METTL3) requires activation of autophagy in human aortic smooth muscle cells (HASMCs). The METTL3 overexpressing HASMCs were infected with lenti‐shATG5, lenti‐shATG7 or lenti‐shRNA, then these HASMCs were used for subsequent experiments. (A) The HASMCs numbers were counted after treated with indicated stimulus (*n* = 4). (B) cell counting kit‐8 (CCK8) assay was used to evaluate cell viability (*n* = 5). (C–F) Representative blots of p‐H3 and PCNA (C,E), and their quantitative results (D,F) in HASMCs (*n* = 4). (G,H) The migration of HASMCs with indicated treatments were detected by using transwell assay (*n* = 15), scale bar: 100 μm. (I–L) The protein levels of MMP2, α‐SMA and SM22a was measured by using western blot in HASMCs. Representative blots (I,K), and their quantitative results (J,L; *n* = 4). β‐Actin serves as loading control. *p < 0.05 versus lenti‐Flag + lenti‐shRNA. #*p <* 0.05 versus lenti‐METTL3 + lenti‐shRNA

More importantly, knockdown of ATG5 or ATG7 dramatically facilitated proliferation, migration and phenotypic switching in METTL3 overexpressing HASMCs (Figure 8A–L). ATG5 or ATG7 knockdown offset the inhibition effects of METTL3 overexpression on proliferation of HASMCs, as evidenced by the following: increased cell numbers (Figure 8A); enhanced cell viability (Figure 8B), and elevated expression levels of proliferation markers, PCNA and p‐H3 (Figure 8C–F). Moreover, ATG5 or ATG7 knockdown significantly abolished the inhibitory effects of METTL3 on migration and synthetic phenotype (Figure 8G–L). Knockdown of ATG5 or ATG7 obviously promoted the migration of HASMCs, and they could largely nullify the inhibitory effect of METTL3 overexpression on HASMC migration (Figure 8G,H), and the expression of MMP2 inhibited by METTL3 was also significantly increased (Figure 8I–L). We also found that the expression of α‐SMA and SM22a was suppressed by knockdown of ATG5 or ATG7 in HASMCs with METTL3 overexpression (Figure 8I–L). Taken together, METTL3‐elicited inhibitory effects on proliferation, migration and phenotypic switching of HASMCs is largely autophagy activation dependent.

## 4 DISCUSSION

**FIGURE 9:**
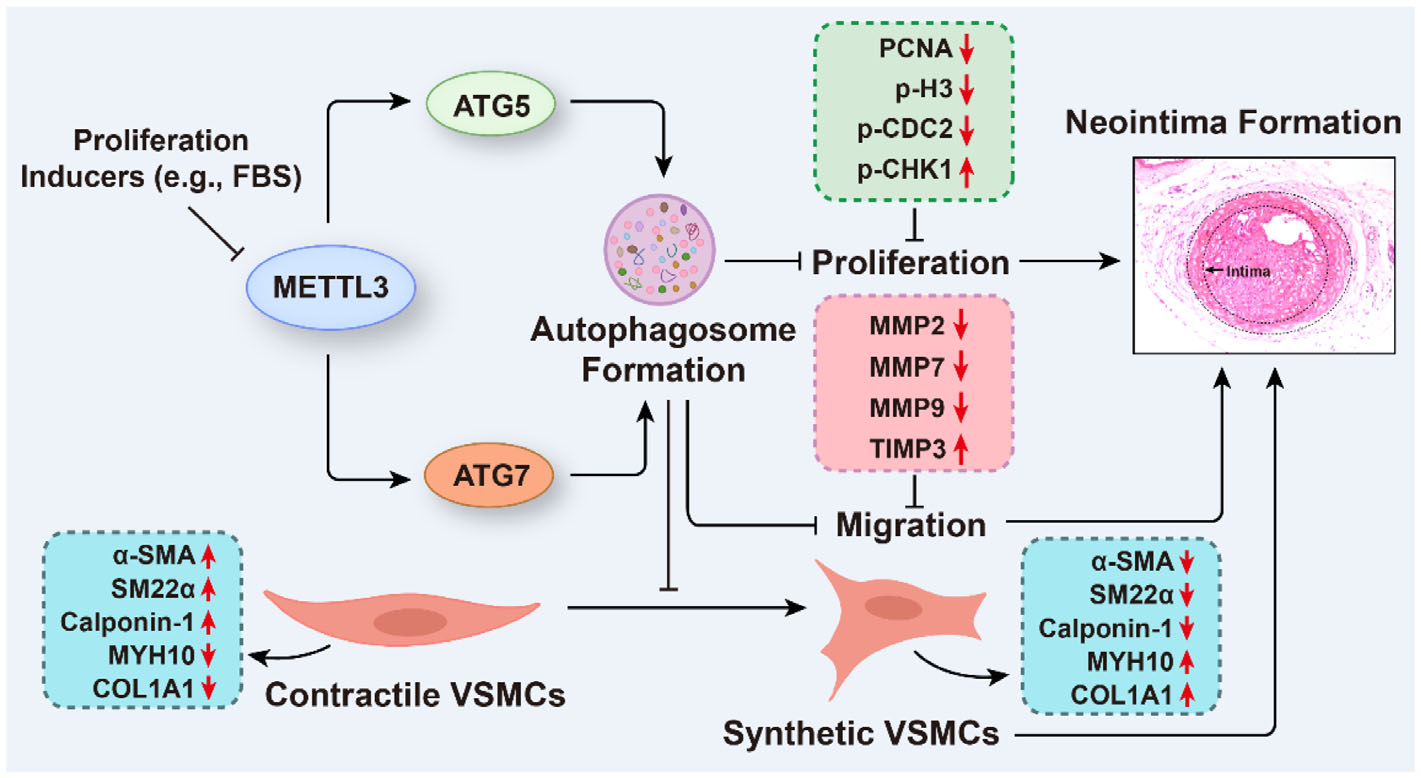
methyltransferase‐like 3 (METTL3) promotes autophagy to inhibit the proliferation, migration and phenotypic switching of vascular smooth muscle cells (VSMCs). The expression of METTL3 was inhibited by proliferation inducers in VSMCs. Overexpression of METTL3 promotes autophagy‐related 5 (ATG5) and ATG7 protein expression to facilitate autophagosome formation, which subsequently inhibits VSMC proliferation, migration and switching from contractile to synthetic phenotype. Knockdown of either ATG5 or ATG7 largely reversed the inhibitory effects of METTL3 on proliferation, migration and phenotypic switching of VSMCs. These findings indicate that METTL3 may inhibit neointima formation by accelerating the formation of autophagosomes.

In this study, we used both gain‐of‐function and loss‐of function approaches to decipher the potential role of METTL3 in proliferation, migration and phenotypic switching of VSMCs (Figure 9). We revealed that the expression level of METTL3 was negatively related to the proliferation of VSMCs and neointima formation. METTL3 knockdown promoted, while METTL3 overexpression inhibited the proliferation, migration and synthetic phenotype of VSMCs. Furthermore, we found that METTL3‐activated autophagy by upregulating ATG5, ATG7 and p‐ULK1, and downregulating p‐mTOR in VSMCs, and knockdown of either ATG5 or ATG7 largely counteracted the effects of METTL3 overexpression on VSMCs. Our results indicated that activation of METTL3 or autophagosome formation may alleviate neointima formation after stenting or coronary artery bypass grafting.

A growing evidence demonstrated that RNA m^6^A modification plays an important role in the regulation of cell proliferation.^29^, ^30^ METTL3, as the most important m^6^A methyltransferase, has also been extensively studied for its effect on cell proliferation, especially in tumour cells, and showed that METTL3 promotes the proliferation and migration of various tumour cells, such as gastrointestinal cancer, bladder cancer, and colorectal cancer.^29^, ^31^ However, the role of METTL3 in VSMC proliferation remains unknown. In the present study, we found that METTL3 knockdown facilitated, while overexpression of METTL3 suppressed the proliferation of VSMCs, which indicated that the role of METTL3 in VSMCs is diametrically opposite to that in tumour cells. The possible reason for this opposite scenario is that there are differences in the energy metabolism of tumour cells and normal cells. Aerobic glycolysis is the energy metabolism of tumour cells, while normal cells use mitochondrial oxidative phosphorylation for energy production.^32^ Similarly, Ma et al.^33^ demonstrated that METTL3 overexpression inhibited the proliferation of retinal pigment epithelial cells through inducing cell cycle arrest at G0/G1 phase. Knockdown of METTL3 promoted the proliferation of human lens epithelial cells induced by high glucose.^34^ METTL3 deficiency also contributed to heart regeneration after myocardial infarction via facilitating cardiomyocytes to re‐enter the cell cycle.^35^ Thus, based on the potential opposed effects of METTL3 on tumour cells and normal cells, if targeting METTL3 or m^6^A to treat tumours, its side effects on normal cells should be carefully considered.

Cell proliferation is controlled by cell cycle checkpoints, and we found that METTL3 arrested HASMCs at G2/M checkpoint by downregulating p‐CDC2 and upregulating p‐CHK1. Binding of CDC2 to Cyclin B1 is required for its activity, which is responsible for entering mitosis.^24^ On the other hand, CHK1 and CHK2 inhibit CDC2 by inactivating the phosphatase CDC25.^24^ It is well known that contractile VSMCs have weak, while synthetic VSMCs have enhanced proliferative and migratory abilities.^36^ METTL3 inhibited the proliferation of VSMCs, implying a phenotypic switching. Our further results revealed that METTL3 knockdown accelerated migration and synthetic phenotype of VSMCs. The loss of contractile properties of smooth muscle cells to a synthetic phenotype is the cause of their hyperproliferation, which will lead to neointima formation.^37^

Although studies have shown that METTL3 has participated in the regulation of autophagy, its role in autophagy remains controversial.^14^, ^15^ In cardiomyocytes, METTL3 has been reported to inhibit autophagy, whereas in NSCLC cells, METTL3 promotes autophagy.^14^, ^15^ In our present study, we found that METTL3‐activated autophagy by upregulating ATG5, ATG7 and p‐ULK1, and downregulating p‐mTOR. Autophagy has been reported to regulate the phenotypic switching of VSMCs, but its effect is context‐dependent.^38^ For example, PDGF‐BB treatment induced VSMC proliferation and synthetic phenotype, as well as autophagy.^39^ In contrast, rapamycin‐based drugs (e.g., sirolimus and everolimus) which were known inhibitors of the mTOR pathway and inducers of autophagy prevented VSMC phenotypic switching and hyperproliferation.^38^, ^40^ Therefore, is there a causal relationship between autophagy and phenotypic switching or a concomitant phenomenon? To answer this question, we reduced autophagosome formation by knocking down ATG5 or ATG7 in METTL3‐overexpressing HASMCs and found that the inhibitory effects of METTL3 on proliferation and migration were significantly reversed by either ATG5 or ATG7 knockdown. Thus, our results indicated that autophagy activation is responsible for attenuated proliferation and migration of VSMCs, at least indispensable for METTL3 overexpression‐mediated maintenance of the contractile phenotype of VSMCs.

METTL3 is the most important RNA m^6^A methyltransferase, which can affect the fate of mRNA by regulating the m^6^A methylation modification of mRNA, such as translation, stability, and splicing.^9^ We found that METTL3 could promote the protein expression of ATG5 and ATG7 to regulate autophagy and phenotypic switching. Many studies have also confirmed that METTL3 usually increases the stability of targeted mRNAs by enhancing their m^6^A modification.^15^, ^41^, ^42^ For example, METTL3 has been reported to promote lung cancer‐associated transcript 3 expression by increasing its mRNA m^6^A level.^42^ We recently reported that METTL3 facilitates ferroptosis of HASMCs by promoting the degradation of SLC7A11 and FSP1 mRNAs.^43^ The limitation of this study is that although we found that METTL3 can increase the protein levels of ATG5 and ATG7, but whether METTL3 directly facilitates the m^6^A of the ATG5 and ATG7 mRNAs and their m^6^A methylation sites are not clear. In addition, the role of METTL3 on neointima formation was not validated by in vivo animal experiments.

In conclusion, we revealed that METTL3 is downregulated during VSMC proliferation, and METTL3 knockdown accelerates the proliferation and migration of VSMCs. Moreover, we further found that METTL3 promotes the formation of autophagosomes, thereby inhibiting the phenotypic switching of VSMCs. These results suggest that activation of autophagy or RNA m^6^A modification may protect against neointima formation after stenting or after coronary artery bypass grafting.

## AUTHOR CONTRIBUTIONS

**Ze‐Min Fang and Shu‐Min Zhang:** designing the study, performing part of experiments, drafting of the article, and providing funding support. **Hanshen Luo, Bo Huo and Yue Chen:** performing cell culture and part of experiments. **Xiaoxuan Zhong, Xin Feng, Wenlin Cheng and Xingliang Wu:** performing analysis and drawing the plots. Gaoke Feng: providing the porcine coronary artery samples. **Ze‐Min Fang,Fang Zhao and Xin Yi:** designing the study, providing funding support and critical revision of the article. **Ding‐Sheng Jiang:** providing writing suggestions and helping revise the article. All authors have read and approved the article for publication.

## CONFLICT OF INTEREST

The authors declare that there is no conflict of interests.

## Data Availability

The data used to support the findings of this study are available from the corresponding author upon request.

## References

[1] Sun LY , Gaudino M , Chen RJ , Bader Eddeen A , Ruel M . Long‐term outcomes in patients with severely reduced left ventricular ejection fraction undergoing percutaneous coronary intervention vs coronary artery bypass grafting. JAMA Cardiol. 2020;5(6):631‐641. 32267465 10.1001/jamacardio.2020.0239 PMC7142806

[2] Gao L , Hu X , Hou Y , Xue Q . Long time clinical outcomes of limus‐eluting stent versus paclitaxel‐eluting stent in patients undergoing percutaneous coronary artery intervention: a meta‐analysis of randomized controlled clinical trials. Cardiol J. 2014;21(3):211‐219. 24526500 10.5603/CJ.a2014.0004

[3] Li R , Yi X , Wei X , et al. EZH2 inhibits autophagic cell death of aortic vascular smooth muscle cells to affect aortic dissection. Cell Death Dis. 2018;9(2):180. 29416002 10.1038/s41419-017-0213-2 PMC5833461

[4] Chen TQ , Hu N , Huo B , et al. EHMT2/G9a inhibits aortic smooth muscle cell death by suppressing autophagy activation. Int J Biol Sci. 2020;16(7):1252‐1263. 32174799 10.7150/ijbs.38835 PMC7053323

[5] Lu W , Zhou Y , Zeng S , et al. Loss of FoxO3a prevents aortic aneurysm formation through maintenance of VSMC homeostasis. Cell Death Dis. 2021;12(4):378. 33828087 10.1038/s41419-021-03659-y PMC8027644

[6] Wei X , Yi X , Zhu XH , Jiang DS . Histone methylation and vascular biology. Clin Epigenetics. 2020;12(1):30. 32070413 10.1186/s13148-020-00826-4 PMC7027016

[7] Wang Y , Xu Y , Yan S , et al. Adenosine kinase is critical for neointima formation after vascular injury by inducing aberrant DNA hypermethylation. Cardiovasc Res. 2021;117(2):561‐575. 32065618 10.1093/cvr/cvaa040 PMC7820850

[8] Li R , Wei X , Jiang DS . Protein methylation functions as the posttranslational modification switch to regulate autophagy. Cell Mol Life Sci. 2019;76(19):3711‐3722. 31222372 10.1007/s00018-019-03161-x PMC11105718

[9] Chen J , Wei X , Yi X , Jiang DS . RNA modification by m(6)a methylation in cardiovascular disease. Oxid Med Cell Longev. 2021;2021:8813909. 34221238 10.1155/2021/8813909 PMC8183103

[10] Chien CS , Li JY , Chien Y , et al. METTL3‐dependent N(6)‐methyladenosine RNA modification mediates the atherogenic inflammatory cascades in vascular endothelium. Proc Natl Acad Sci U S A. 2021;118(7):e2025070118. 33579825 10.1073/pnas.2025070118 PMC7896299

[11] Dong G , Yu J , Shan G , Su L , Yu N , Yang S . N6‐Methyladenosine methyltransferase METTL3 promotes angiogenesis and atherosclerosis by upregulating the JAK2/STAT3 pathway via m6A reader IGF2BP1. Front Cell Dev Biol. 2021;9:731810. 34950654 10.3389/fcell.2021.731810 PMC8689138

[12] Zhong L , He X , Song H , et al. METTL3 induces AAA development and progression by modulating N6‐Methyladenosine‐dependent primary miR34a processing. Mol Ther Nucleic Acids. 2020;21:394‐411. 32650237 10.1016/j.omtn.2020.06.005 PMC7347714

[13] Wang G , Dai Y , Li K , et al. Deficiency of Mettl3 in bladder cancer stem cells inhibits bladder cancer progression and angiogenesis. Front Cell Dev Biol. 2021;9:627706. 33681207 10.3389/fcell.2021.627706 PMC7930389

[14] Song H , Feng X , Zhang H , et al. METTL3 and ALKBH5 oppositely regulate m(6)a modification of TFEB mRNA, which dictates the fate of hypoxia/reoxygenation‐treated cardiomyocytes. Autophagy. 2019;15(8):1419‐1437. 30870073 10.1080/15548627.2019.1586246 PMC6613905

[15] Liu S , Li Q , Li G , et al. The mechanism of m(6)a methyltransferase METTL3‐mediated autophagy in reversing gefitinib resistance in NSCLC cells by beta‐elemene. Cell Death Dis. 2020;11(11):969. 33177491 10.1038/s41419-020-03148-8 PMC7658972

[16] Grootaert MO , da Costa Martins PA , Bitsch N , et al. Defective autophagy in vascular smooth muscle cells accelerates senescence and promotes neointima formation and atherogenesis. Autophagy. 2015;11(11):2014‐2032. 26391655 10.1080/15548627.2015.1096485 PMC4824610

[17] Ouyang C , Li J , Zheng X , et al. Deletion of Ulk1 inhibits neointima formation by enhancing KAT2A/GCN5‐mediated acetylation of TUBA/alpha‐tubulin in vivo. Autophagy. 2021;17(12):4305‐4322. 33985412 10.1080/15548627.2021.1911018 PMC8726707

[18] Chen Y , Wei X , Zhang Z , et al. Downregulation of filamin a expression in the aorta is correlated with aortic dissection. Front Cardiovasc Med. 2021;8:690846. 34485398 10.3389/fcvm.2021.690846 PMC8414519

[19] Yi X , Zhou Y , Chen Y , et al. The expression patterns and roles of Lysyl oxidases in aortic dissection. Front Cardiovasc Med. 2021;8:692856. 34307505 10.3389/fcvm.2021.692856 PMC8292648

[20] Chen Y , Yi X , Huo B , et al. BRD4770 functions as a novel ferroptosis inhibitor to protect against aortic dissection. Pharmacol Res. 2022;177:106122. 35149187 10.1016/j.phrs.2022.106122

[21] Chen YJ , Li Y , Guo X , et al. Upregulation of IRF9 contributes to pulmonary artery smooth muscle cell proliferation during pulmonary arterial hypertension. Front Pharmacol. 2021;12:773235. 34925032 10.3389/fphar.2021.773235 PMC8672195

[22] Feng G , Xiao J , Bi Y , et al. 12‐month coronary angiography, intravascular ultrasound and histology evaluation of a novel fully bioabsorbable poly‐L‐lactic acid/amorphous calcium phosphate scaffolds in porcine coronary arteries. J Biomed Nanotechnol. 2016;12(4):743‐752. 27301200 10.1166/jbn.2016.2241

[23] Xiao J , Feng G , Kang G , et al. 6‐month follow‐up of a novel biodegradable drug‐eluting stent composed of poly‐L‐lactic acid and amorphous calcium phosphate nanoparticles in porcine coronary artery. J Biomed Nanotechnol. 2015;11(10):1819‐1825. 26502644 10.1166/jbn.2015.2102

[24] Taylor WR , Stark GR . Regulation of the G2/M transition by p53. Oncogene. 2001;20(15):1803‐1815. 11313928 10.1038/sj.onc.1204252

[25] Vera J , Raatz Y , Wolkenhauer O , et al. Chk1 and Wee1 control genotoxic‐stress induced G2‐M arrest in melanoma cells. Cell Signal. 2015;27(5):951‐960. 25683911 10.1016/j.cellsig.2015.01.020

[26] Miano JM , Fisher EA , Majesky MW . Fate and state of vascular smooth muscle cells in atherosclerosis. Circulation. 2021;143(21):2110‐2116. 34029141 10.1161/CIRCULATIONAHA.120.049922 PMC8162373

[27] Mittal B , Mishra A , Srivastava A , Kumar S , Garg N . Matrix metalloproteinases in coronary artery disease. Adv Clin Chem. 2014;64:1‐72. 24938016 10.1016/b978-0-12-800263-6.00001-x

[28] Lu QB , Wan MY , Wang PY , et al. Chicoric acid prevents PDGF‐BB‐induced VSMC dedifferentiation, proliferation and migration by suppressing ROS/NFkappaB/mTOR/P70S6K signaling cascade. Redox Biol. 2018;14:656‐668. 29175753 10.1016/j.redox.2017.11.012 PMC5716955

[29] Wang Q , Geng W , Guo H , et al. Emerging role of RNA methyltransferase METTL3 in gastrointestinal cancer. J Hematol Oncol. 2020;13(1):57. 32429972 10.1186/s13045-020-00895-1 PMC7238608

[30] Yang B , Wang JQ , Tan Y , Yuan R , Chen ZS , Zou C . RNA methylation and cancer treatment. Pharmacol Res. 2021;174:105937. 34648969 10.1016/j.phrs.2021.105937

[31] Lan Q , Liu PY , Bell JL , et al. The emerging roles of RNA m(6)a methylation and demethylation as critical regulators of tumorigenesis, drug sensitivity, and resistance. Cancer Res. 2021;81(13):3431‐3440. 34228629 10.1158/0008-5472.CAN-20-4107

[32] Vander Heiden MG , Cantley LC , Thompson CB . Understanding the Warburg effect: the metabolic requirements of cell proliferation. Science. 2009;324(5930):1029‐1033. 19460998 10.1126/science.1160809 PMC2849637

[33] Ma X , Long C , Wang F , et al. METTL3 attenuates proliferative vitreoretinopathy and epithelial‐mesenchymal transition of retinal pigment epithelial cells via wnt/beta‐catenin pathway. J Cell Mol Med. 2021;25(9):4220‐4234. 33759344 10.1111/jcmm.16476 PMC8093987

[34] Yang J , Liu J , Zhao S , Tian F . N(6)‐Methyladenosine METTL3 modulates the proliferation and apoptosis of lens epithelial cells in diabetic cataract. Mol Ther Nucleic Acids. 2020;20:111‐116. 32163892 10.1016/j.omtn.2020.02.002 PMC7066033

[35] Gong R , Wang X , Li H , et al. Loss of m(6)a methyltransferase METTL3 promotes heart regeneration and repair after myocardial injury. Pharmacol Res. 2021;174:105845. 34428587 10.1016/j.phrs.2021.105845

[36] Chen LD , Zhu WT , Cheng YY , et al. T‐cell death‐associated gene 8 accelerates atherosclerosis by promoting vascular smooth muscle cell proliferation and migration. Atherosclerosis. 2020;297:64‐73. 32078831 10.1016/j.atherosclerosis.2020.01.017

[37] Gong X , Tian M , Cao N , et al. Circular RNA circEsyt2 regulates vascular smooth muscle cell remodeling via splicing regulation. J Clin Invest. 2021;131(24):e147031. 34907911 10.1172/JCI147031 PMC8670847

[38] Salabei JK , Hill BG . Implications of autophagy for vascular smooth muscle cell function and plasticity. Free Radic Biol Med. 2013;65:693‐703. 23938401 10.1016/j.freeradbiomed.2013.08.003 PMC3859773

[39] Salabei JK , Cummins TD , Singh M , Jones SP , Bhatnagar A , Hill BG . PDGF‐mediated autophagy regulates vascular smooth muscle cell phenotype and resistance to oxidative stress. Biochem J. 2013;451(3):375‐388. 23421427 10.1042/BJ20121344 PMC4040966

[40] Marx SO , Jayaraman T , Go LO , Marks AR . Rapamycin‐FKBP inhibits cell cycle regulators of proliferation in vascular smooth muscle cells. Circ Res. 1995;76(3):412‐417. 7532117 10.1161/01.res.76.3.412

[41] Jin D , Guo J , Wu Y , et al. M(6)a mRNA methylation initiated by METTL3 directly promotes YAP translation and increases YAP activity by regulating the MALAT1‐miR‐1914‐3p‐YAP axis to induce NSCLC drug resistance and metastasis. J Hematol Oncol. 2019;12(1):135. 31818312 10.1186/s13045-019-0830-6 PMC6902496

[42] Qian X , Yang J , Qiu Q , et al. LCAT3, a novel m6A‐regulated long non‐coding RNA, plays an oncogenic role in lung cancer via binding with FUBP1 to activate c‐MYC. J Hematol Oncol. 2021;14(1):112. 34274028 10.1186/s13045-021-01123-0 PMC8285886

[43] Li N , Yi X , He Y , et al. Targeting ferroptosis as a novel approach to alleviate aortic dissection. Int J Biol Sci. 2022;18(10):4118‐4134. 35844806 10.7150/ijbs.72528 PMC9274489

